# What underlies waves of agitation in starling flocks

**DOI:** 10.1007/s00265-015-1891-3

**Published:** 2015-03-25

**Authors:** Charlotte K. Hemelrijk, Lars van Zuidam, Hanno Hildenbrandt

**Affiliations:** Behavioural Ecology and Self-organisation, Groningen Institute for Evolutionary Life Sciences, University of Groningen, Nijenborgh 7, 9747AG Groningen, The Netherlands

**Keywords:** Agitation wave in a starling flock, Escape manoeuvre, Wave speed, Information transmission, Individual-based model, Collective motion

## Abstract

Fast transfer of information in groups can have survival value. An example is the so-called wave of agitation observed in groups of animals of several taxa under attack. It has been shown to reduce predator success. It usually involves the repetition of a manoeuvre throughout the group, transmitting the information of the attack quickly, faster than the group moves itself. The specific manoeuvre underlying a wave is typically known, but not so in starlings (*Sturnus vulgaris*). Although waves of agitation in starling flocks have been suggested to reflect density waves, exact escape manoeuvres cannot be distinguished because flocks are spatially too far away. Therefore, waves may also reflect orientation waves (due to escape by rolling). In the present study, we investigate this issue in a computational model, StarDisplay. We use this model because its flocks have been shown to resemble starling flocks in many traits. In the model, we show that agitation waves result from changes in orientation rather than in density. They resemble empirical data both qualitatively in visual appearance and quantitatively in wave speed. In the model, local interactions with only two to seven closest neighbours suffice to generate empirical wave speed. Wave speed increases with the number of neighbours mimicked or repeated from and the distance to them. It decreases with reaction time and with time to identify the escape manoeuvre of others and is not affected by flock size. Our findings can be used as predictions for empirical studies.

**Electronic supplementary material:**

The online version of this article (doi:10.1007/s00265-015-1891-3) contains supplementary material, which is available to authorized users.

## Introduction

Fast transfer of information in groups can have survival value (Krause and Ruxton 2002). When swarms of animals (be it insects, fish, or birds) are under attack of a predator, fast information transfer as in the so-called waves of agitation or shimmering waves (Radakov 1973; Treherne and Foster 1981; Axelsen et al. 2001; Gerlotto et al. 2006; Kastberger et al. 2008; Procaccini et al. 2011), is associated with reduced catch rate of the predator (Treherne and Foster 1982; Kastberger et al. 2008; Procaccini et al. 2011).

These waves may reveal themselves as spirals, concentric rings or moving lines. They are caused by individuals repeating a fear reaction or escape manoeuvre of a neighbour close by (Gerlotto et al. 2006). The transferred manoeuvre differs between species: in giant honeybees individuals lift their abdomen, in ocean skaters individuals perform fast random movements (Treherne and Foster 1982; Kastberger et al. 2008) and in fish and birds individuals move closer together (Axelsen et al. 2001; Procaccini et al. 2011) or roll sideward (Radakov 1973; Potts 1984; Gerlotto et al. 2006). Agitation waves move faster than some or all of the following factors: the average speed of movement of the group (Radakov 1973; Gerlotto et al. 2006; Procaccini et al. 2011), the speed of attack by the predator (Radakov 1973; Treherne and Foster 1982; Marras et al. 2012) and the speed resulting from individuals transferring information to their closest neighbours delayed only by their reaction time (Kastberger et al. 2008). Some kind of long-range anticipation has been deemed to be necessary for generating such speed. It was thought to involve anticipation of the approach of the wave-front from a larger distance than the nearest neighbours (the so-called chorus line hypothesis) (Potts 1984) or to involve transmission by jumps to more distant individuals than closeby neighbours, individuals that were supposed to be specialised in responding (Kastberger et al. 2012).

Recently, a study of the remarkable waves of agitation in starling flocks (*Sturnus vulgaris*) has shown that the dark band moves continuously in a line from one end of the flock to the other end always away from the predator (Procaccini et al. 2011). Waves are presumed to involve individuals that are moving closer together and further apart in so-called density waves (Procaccini et al. 2011). However, starling flocks are actually too far away from the observer to identify what the transferred manoeuvre is (Procaccini et al. 2011). Possibly, individuals do not repeat a specific manoeuvre at all and merely adjust their movement direction and speed to others. Alternatively, they may use a specific escape manoeuvre. Here, we may distinguish two types of manoeuvres: those that lead to a density wave and those that lead to an orientation wave. For instance, individuals in the flock that move away from the predator fast may increase the local density of the flock temporarily (generating a density wave) and individuals that change direction by rolling sideward may generate an orientation wave. Waves of such escape reactions by changes in orientation are observed in flocks of dunlins and in schools of anchovies. Here, rolling sideward by individuals is accompanied by changes in the colour of the swarm; in dunlins between brown (dorsal side of the bird) and white (its belly) (Potts 1984; Buchanan et al. 1988) and in schools of anchovy between dark (dorsal part of fish) and silvery (belly of the fish) (Radakov 1973; Gerlotto et al. 2006). In starlings, there are no such colour differences between the dorsal and ventral side of the body. We speculate that orientation waves in their case, instead, arise from a difference in surface area of the wing becoming visible to the observer when the starling rolls sideward or not.

As to explaining the speed of the wave of starlings, waves move on average at a speed of 13.4 m/s (Procaccini et al. 2011). Thus, waves move faster than the flock itself, namely on average at 10.6 m/s (Ballerini et al. 2008b). Wave speed is close to the speed of the predator, which is between 11 and 15 m/s (Cornell lab of ornithology). Yet, no long distance anticipation is needed, because wave speed is close to the value of the quotient of the average distance to nearest neighbour of 1.1 m (Ballerini et al. 2008b) divided by reaction time of others of 0.076 s (Pomeroy and Heppner 1977), namely 14.5 m/s. Thus, wave speed may arise from transfer between the nearest neighbours in a line, called bucket brigade (Kastberger et al. 2012).

Because empirical observation of the escape manoeuvre of these waves is not yet possible, in the present paper, we use a computational model of starling flocks, StarDisplay (Hildenbrandt et al. 2010; Hemelrijk and Hildenbrandt 2011), to infer what type of manoeuvre (if at all) may underlie the wave and what factors cause high wave speed. StarDisplay is the right framework for this examination, for several reasons. First, it includes simplified flying behaviour next to the rules for coordination by attraction, alignment and avoidance as used for studying fish schools (Huth 1992; Kunz and Hemelrijk 2003; Hemelrijk and Hildenbrandt 2008). Flying behaviour is shown to be essential for generating the variation of flock shapes resembling empirical data (Pomeroy and Heppner 1992; Hemelrijk and Hildenbrandt 2012). Second, its biologically inspired rules are tuned to biologically relevant parameters. Third, its patterns of flocking resemble empirical data in many ways, as regards (a) shape and orientation of the flock; (b) aspects of turning, such as maintenance of shape during a turn, the change of the orientation of the shape relative to the movement direction and the repositioning of individuals during turns as well as (c) aspects of internal structure, namely as measured by the scale free correlation between the absolute length of the flock (in m) and the correlation length of the deviation of the velocity and speed of individuals from that of the centre of gravity also in relation to speed control (Hemelrijk and Hildenbrandt 2012, 2015; Bialek et al. 2014) as by the degree of disorder or diffusion in the group (CKH and HH, unpublished data).

In the model, we first explore whether an agitation wave emerges when no escape manoeuvre is repeated, and if not, we investigate, the repetition of which manoeuvre causes it. Note that not all imaginable escape manoeuvres may underlie agitation waves. For instance, in models of fish schools, ‘moving away’ from the predator has been shown to change the shape of the school in bend, flash expansion, vacuoles *etcetera* and may even split the school into sub-groups (Inada and Kawachi 2002). This cannot underlie the agitation wave because during an agitation wave the flock maintains its shape (Procaccini et al. 2011). Therefore, we investigated two new types of escape manoeuvres (not studied in models of coordinated grouping before) that are observed in birds and approximately preserve flock shape. A manoeuvre possibly underlying a density wave, namely speeding up forwards in the flock away from the predator (Procaccini et al. 2011), and a manoeuvre that may underlie an orientation wave, namely rolling sideward and back like in a zigzag (Rudebeck 1951).

As factors affecting wave speed, we experiment with distance to the nearest neighbours, the number of neighbours whose escape manoeuvre is mimicked or repeated (from now on called, the range of repetition), reaction time, cue identification time (for repeating an escape signal) and flock size. We compare wave speeds to those reported for empirical data (Procaccini et al. 2011).

## Methods

We extended our model, StarDisplay, with two types of escape reactions and a transmission mechanism. We performed our experiments of escape in this extended model.

### The model

#### General outline

**Fig. 1:**
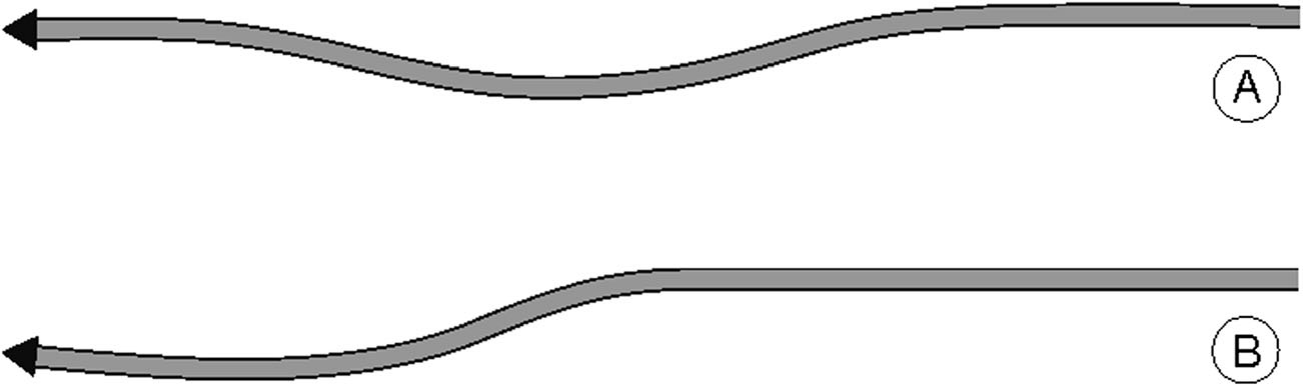
Trajectory of a bird when escaping by **a** a zigzag-manoeuvre with two turns or **b** a zig-manoeuvre comprising a single turn to the left. *Black arrowhead* represents the individual bird

Because flying implies movement in all directions, we developed our model in three dimensions. The behaviour of each individual in StarDisplay is based on its cruise speed, its social coordination (depending on the position and heading of its nearby neighbours), its attraction to the roost (site for sleeping), the simplified aerodynamics of flight which includes banking while turning, and reaction time (Hemelrijk and Hildenbrandt 2011). One of the sources of error is that we update the location and heading of each individual at shorter intervals than the interval of the reaction time. For other sources of error, see the random error (Equ S14) in the description of the model in the supplementary material and in our former work (Hemelrijk and Hildenbrandt 2011). The results of the model are robust against such sources of random error. We model social coordination in terms of (social) forces in line with studies by others (Helbing and Molnar 1995; Couzin et al. 2002; Hemelrijk and Hildenbrandt 2008). In the present paper, we have added two types of escape manoeuvre that preserved flock shape: 1) the zigzag-manoeuvre and its halved version, a ‘zig’-manoeuvre of rolling sideward and back (Fig. 1) involving a change of orientation and 2) a manoeuvre involving an acceleration forward in the flock (‘speeding-up-forward’) causing a change of density.

Further, we made flying more natural by increasing the tendency of individuals to pitch and representing head nystagmus as is observed in real birds. Head nystagmus implies that birds stabilise their visual system by keeping their heads still, while banking their body sideward (Warrick et al. 2002). For this, we modelled the head system separately from the body system.

During their normal reaction time of 76 ms (Pomeroy and Heppner 1977) (Table 1), birds do not update their environment while they are flying still. Therefore, in the model this is an important cause of error in their behavioural response.

**Table 1:** Parameters of escape reactions in the model

Parameter	Description	Default	Experimental values
*N*	Flock size	2000 indiv.	500, 1000, 2000, 4000, 8000
*Δu*	Average reaction time	0.076 s	(Pomeroy and Heppner 1977)
*σ*_*u*_	Standard deviation reaction time	0.01 s	
NND	Nearest neighbour distance	1.3 m	0.73, 0.93, 1.13, 1.32, 1.52, 1.74,1.94
*r*_sep_	Separation radius	2 m	1.0, 4/3, 5/3, 2.0, 7/3, 8/3, 3.0
*topo*	Number of influential neighbours	6–7 neighb.	
Range_Rep_	Repetition range	6 neighb.	1, 2, 3, 4, 5, 6, 7
*T*_cue identification_	Cue identification time to recognise an escape manoeuvre	0.05 s	
*T*_zside_	Evasion time to zig sidewards	0.25 s	
*T*_zback_	Evasion time to zig back	0.30 s	
*T*_sf_	Evasion time to speed-up-forward	0.5 s	
*T*_rp_	Duration refractory period	1.00 s	
*w*_zig_	Weight zig	1 N	
*wsf*	Weight speed-up-forward	1 N	
wa_*h*_	Weighting factor alignment force heading	1 N	
wa_*b*_	Weighting factor alignment force banking	2 N	
	Acclimation time of simulation	50 s	

We use SI units and choose real parameter values where available (see Parameterization and Suppl. material, Table S1). For details of the model and the basic behavioural rules, see Supplementary material.

#### Initial condition, escape behaviour and computational experiments

In our earlier simulations, the attraction to return to the site for sleeping (roost) induced many turns of the flock (Hildenbrandt et al. 2010; Hemelrijk and Hildenbrandt 2011, 2012). For studying the agitation wave, we want a flock that does not change its shape and, therefore, does not turn. Therefore, we omitted the attraction to a roost by using a roost or sleeping site of infinite size. The simulation started with a single flock of randomly positioned individuals in a small volume of space, at an approximately default average distance to the nearest neighbours. In order for the normal flocking behaviour to emerge, data collection started after an acclimatisation period of 50 s (Table 1).

Note that the surprise attack is the most common attack strategy of falcons on flocks of starlings (Rudebeck 1950; Zoratto et al. 2010). This is the attack we used.

Since it made no difference in speed of transmission whether we attacked individuals at the rear end of the flock, the side or the front of it, we confined ourselves to attacks from the back because flocks may often face away from the predator when under attack. We need to find individuals at the rear in the model, which is setup in a Euclidian 3D space (based on three perpendicular axes: x, y, and z). The location of the body of each individual relative to the origin is indicated by a vector ***p***. The orientation of the body is given by its forward direction, ***e***_***x***_, its sideward direction, ***e***_***y***_, and its upward direction, ***e***_***z***_, which may change by rotating around these three principal axes, ***e***_***x***_, ***e***_***y***_ and ***e***_***z***_ (*roll*, *pitch* and *yaw*) (Fig. S2). The individual *i* at the back of the flock, *i*_rear_ (Eq. 3), is found by the lowest value of the dot product between the position of each individual relative to the centre of gravity of the flock (which is the average position of all flock members, Eq. 1) and the average direction of movement of the flock (Eq. 2).

1 $$ \overline{p}=\frac{1}{N}{\displaystyle {\sum}_i}{\boldsymbol{p}}_i\kern2.12em \mathrm{Flock}\kern0.5em \mathrm{centre}\kern0.5em \mathrm{of}\kern0.5em \mathrm{gravity} $$

2 $$ \overline{e_x}={\displaystyle {\sum}_i}{\boldsymbol{e}}_{\boldsymbol{xi}}/\left|{\displaystyle {\sum}_i}{\boldsymbol{e}}_{\boldsymbol{xi}}\right|\kern1.12em \mathrm{Direction}\ \mathrm{of}\ \mathrm{flock}\kern0.5em \mathrm{movement} $$

3 $$ {i}_{\mathrm{rear}}=\left\{i\in N;\ {\boldsymbol{p}}_i\overline{e_x}\kern0.5em \mathrm{is}\kern0.5em \mathrm{minimal}\right\}\kern1.12em \mathrm{Hindmost}\kern0.5em \mathrm{individual} $$

where *N* is the number of individuals in the flock, ***p***_***i***_ indicates the position of individual *i*, and ***e***_***xi***_ represents the forward direction of individual *i*.

We made the hindmost individual, *i*_rear_, escape by one of the three escape manoeuvres, sidewards and back, namely zigzag and zig, or accelerate forward (called speed-up-forward). Because the social coordination (Supplementary material, Equ S5–S12) was modelled by us and others based on social forces (Helbing and Molnar 1995; Couzin et al. 2002; Hemelrijk and Hildenbrandt 2008), we used social forces for the escape manoeuvres also.

In case of the zigzag-manoeuvre, the individual moves sideward (by rolling), back and sideward to the other side again (Fig. 1a).

4 $$ \begin{array}{ll}{\boldsymbol{f}}_{\mathrm{zz}}={w}_{\mathrm{zig}}{\boldsymbol{e}}_{\boldsymbol{y}};\hfill & \hfill 0<t<{T}_{\mathrm{zside}}\hfill \\ {}{\boldsymbol{f}}_{\mathrm{zz}}=-{w}_{\mathrm{zig}}{\boldsymbol{e}}_{\boldsymbol{y}};\hfill & \hfill {T}_{\mathrm{zside}}<t<{T}_{\mathrm{zside}}+{T}_{\mathrm{zback}}\hfill \\ {}{\boldsymbol{f}}_{\mathrm{zz}}={w}_{\mathrm{zig}}{\boldsymbol{e}}_{\boldsymbol{y}};\hfill & {T}_{\mathrm{zside}}+{T}_{\mathrm{zback}}<t<{T}_{\mathrm{zside}}+{T}_{\mathrm{zback}}+{T}_{\mathrm{zside}}\hfill \end{array} $$

where ***f***_zz_ is the force. As a consequence of it, the bird moved *T*_zside_ seconds to the side and *T*_zback_ seconds back again and again *T*_zside_ seconds to the other side and *T*_zback_ seconds back again (Table 1).

The zig-manoeuvre represents only half of the zigzag, thus, rolling sideward *T*_zside_ seconds and *T*_zback_ seconds back again (Table 1). This causes a small sideward shift of the individual to the left (Fig. 1b).

The manoeuvre of speeding-up-forward is modelled by

5 $$ {\boldsymbol{f}}_{\mathrm{sf}}={w}_{\mathrm{sf}}{\boldsymbol{e}}_{\boldsymbol{x}};\kern2em 0<t < {T}_{\mathrm{sf}} $$

and involves the force ***f***_sf_ that causes the individual to accelerate for *T*_sf_ seconds forwards (Table 1). After each escape event, the individual recovered during a short refractory period of *T*_rp_ seconds (Table 1).

We investigated whether transmission of information about escape in the model happened either by individual adjustment of their movement to a close-by escape manoeuvre of another individual or happened by individual recognition of the escape manoeuvre followed by repeating it (Potts 1984). Such recognition and identification of an escape manoeuvre takes time, which we called cue identification time *T*_cue_. In line with studies of others (Bode et al. 2010), we assumed this cue identification time to be shorter than the normal reaction time. The number of neighbours that an individual scanned for a potential escape manoeuvre is labelled as the range of repetition, Range_Rep_ (Table 1).

#### Parameterization

**Fig. 2:**
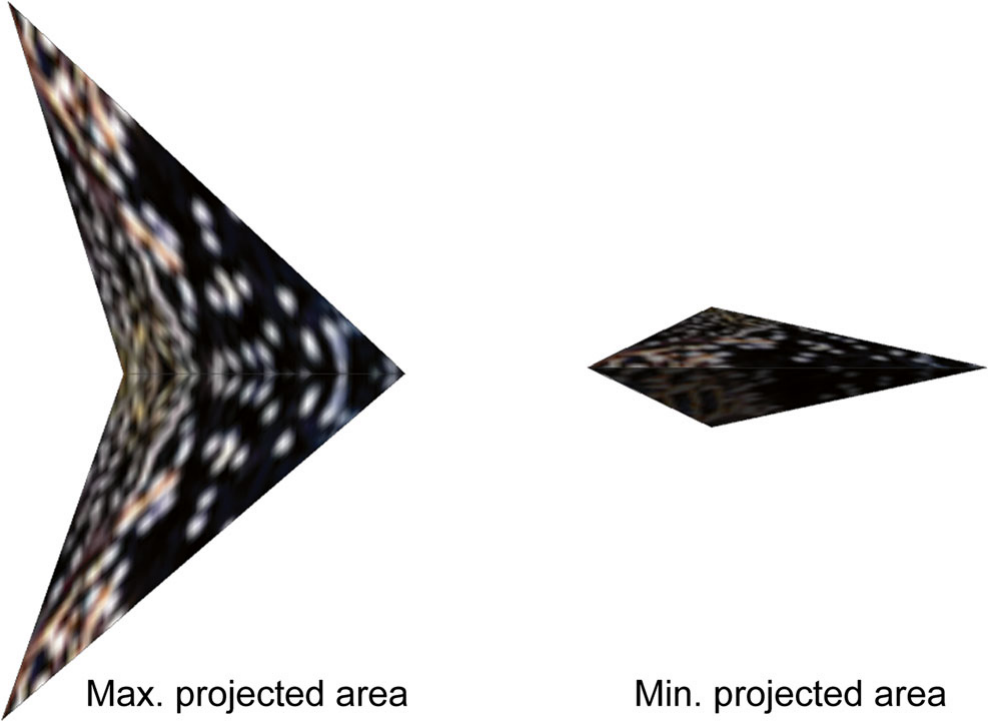
Representation of the bird’s projected area in the model for two different views (seen from the side): the maximum projected area when the bird rolls maximally, and minimum projected area when the bird flies level

We represented birds in the model by an ‘arrowhead’ of similar aspect ratios of wing span versus length and height as the starling (Fig. 2a, Table S1) (Videler 2005).

We have parameterized individuals in the model to realistic data of birds (weight, cruise speed, etcetera), especially of starlings, see supplementary material Table S1 and our earlier version of StarDisplay (Hildenbrandt et al. 2010; Hemelrijk and Hildenbrandt 2011). Roll rate and banked turns were tuned to those observed in movies of starlings in that they rolled into the turn faster than that they rolled back (Gillies et al. 2011), roll rate is within the range measured for other species (Gillies et al. 2008, 2011) and banked turns resemble empirical data in that individuals lose height during turns (Pomeroy and Heppner 1992; Gillies et al. 2011).

**Fig. 3:**
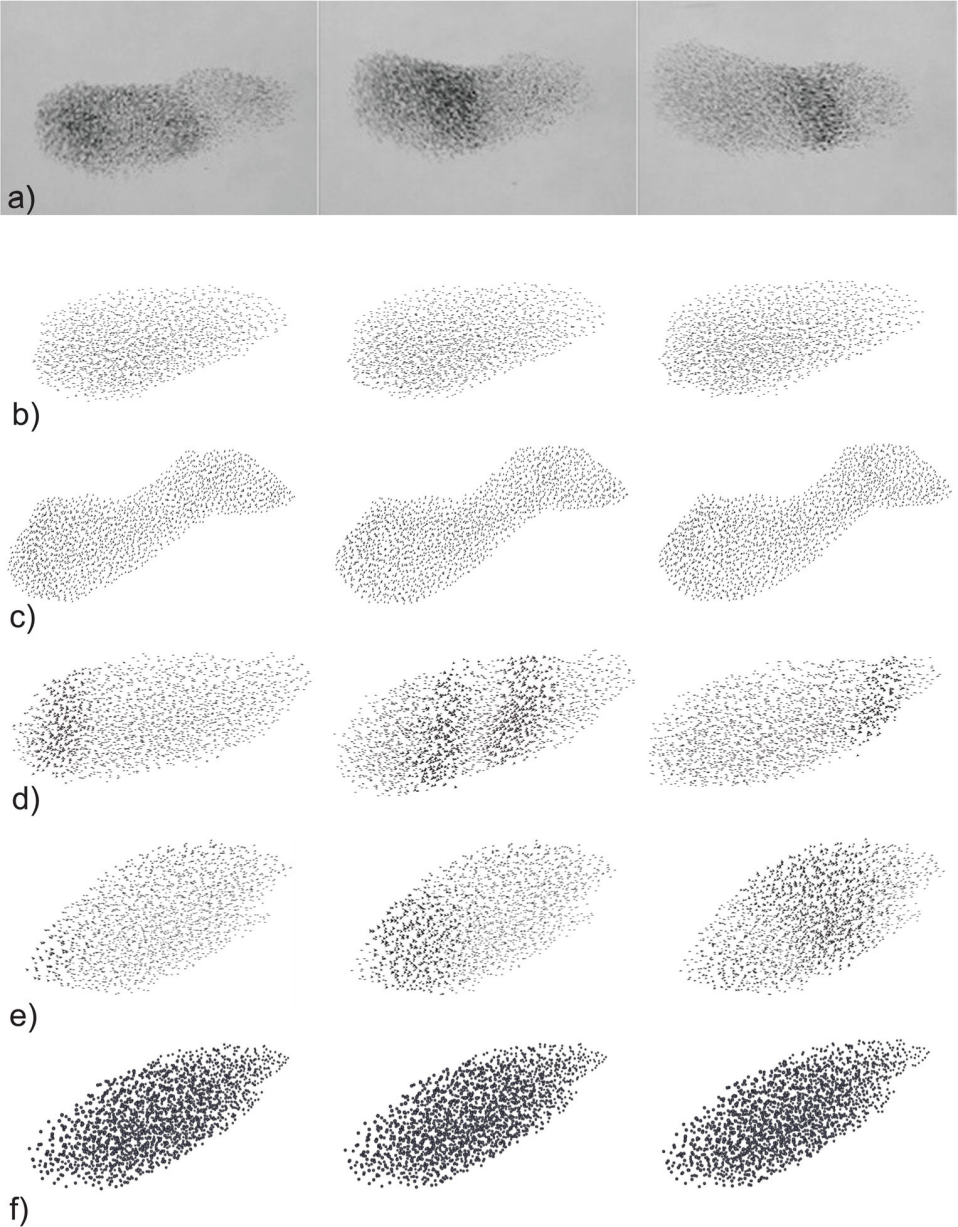
Three subsequent images (*left to right*) of a flock being attacked at the left side of the pictures: (**a**) in empirical data (from C. Carere) and (**b**–**f**) the model. This shows (**a**) the wave in the empirical data; (**b**) absence of a wave if the escape manoeuvre (a zig) is not repeated; (**c**) absence of a wave if individuals repeat the escape manoeuvre of speeding-up-forward; (**d**) a double-banded wave if the birds repeat the zigzag-escape of rolling to one side, back, rolling to the other side and back again; (**e**) a single-banded wave if the escape manoeuvre involves rolling only to one sided and back (displaying a zig); (**f**) and absence of a wave if body shape is spherical while birds escape by rolling sideward and back, thus executing a zig-manoeuvre. See also movies S1–S5. Note that speed-forward is observed from below and the rolling movements are observed from the side

Because agitation waves have particularly been observed in flocks of large sizes (Procaccini et al. 2011), as a default flock size we used 2000 individuals (Fig. 3) (Ballerini et al. 2008b) with an average distance to their nearest neighbours of 1.3 m resembling empirical data (Major and Dill 1978; Ballerini et al. 2008b). When an individual observed in its range of repetition another individual displaying an escape manoeuvre, it was made to repeat this manoeuvre. We choose a topological range of six to seven closest neighbours to repeat the escape manoeuvre from because this is also the topological range observed empirically during coordination in a flock in the absence of predation (Ballerini et al. 2008a). Here, the topological range included all six to seven nearest neighbours outside the blind angle at the back (Table S1). Empirically, in large-scale stereometric analyses, this number has been established during normal coordination in a flock (in the absence of a predator) as being the number of influential neighbours (Ballerini et al. 2008a). As to the average reaction time during coordination, we used the only empirical data available of 76 ms, which concerns the startle reaction to a light stimulus (Pomeroy and Heppner 1977). We represented variation in reaction time by drawing values from a normal distribution with a standard deviation *σ*_*u*_ of 10 ms (Table 1). The shortest time needed to recognise an escape manoeuvre is 0.05 s after its start, a value which was inspired by measurements of fish to react to a predator threat (Domenici and Batty 1997). This delay, we label the cue identification time (Table 1). The actual start of the repetition of the escape manoeuvre depends also on the reaction interval (of 0.076 s). Thus on average, individuals repeated an escape manoeuvre after 0.05 s + (0.076/2) = 0.043 s.

When studying flock size (between 500 and 8000 individuals, see Table 1), reaction time and cue identification time, we kept the distance to the nearest neighbours constant by adjusting the separation radius, *r*_sep_ (Table 1) (Hildenbrandt et al. 2010; Hemelrijk and Hildenbrandt 2015). To investigate the effects of the density of the flock (measured as average distance to the nearest neighbours, NND), we tuned density with the separation radius, *r*_sep_ (Table 1).

#### Observations and measurements

To detect potential waves of agitation, we have recorded in the model with a virtual camera the flock from the side and from below resembling the setting of the real camera in the empirical study (Procaccini et al. 2011).

We measured the wave speed in the model by starting from the time that the wave has arrived at the centre of gravity of the flock (and is thus clearly visible) by calculating for each escaping individual its spatial distance to the first bird escaping and the time interval between the escape of the first individual and itself. The wave speed is the average of all these measurements. We took the average rather than the median because the variation in reaction time has been drawn from a normal distribution.

For each parameter value of NND, range of repetition, reaction time, cue identification time and flock size, we have run 30 replicas.

## Results

In empirical studies of flocks of starlings, an agitation wave has been described as a dark band moving away from the attack by the predator to the other side of the flock (Fig. 2a).

Our model did not produce an agitation wave, if the flock members merely adjusted their movement but did not repeat an escape manoeuvre (Fig. 3b movie S1). Nor did a wave emerge if individuals repeated the escape manoeuvre of speeding up forward (Fig. 3c). Repetition of speeding-up-forward merely creates some extra movement in the flock (Movie S2). Repeated escape manoeuvres produced an agitation wave, in the form of double dark bands moving away from the predator to the other side of the flock (Fig. 3d, Movie S3) if the prey reacted by repeating the specific zigzag-manoeuvre. To obtain a single band, the escape manoeuvre needed to be halved; thus, individuals are rolling sideward and back again, thus displaying half a ‘zigzag’ which we name a ‘zig’ (Fig. 3e, movie S4a b).

The zig movement generated an agitation wave merely because temporarily rolling laterally changed the orientation of the birds towards the observer which implies a difference in the visibility of the wing area (projected area, see Fig. 2a). If the observer is located to the side, the observer will see the largest wing area once a bird has rolled 90° sidewards. This temporary increase of dark surface of the wing causes us to see a black band, moving away from the predator. Yet the behavioural rule of the zig-escape still may have also an effect on the density in the flock. To investigate this, we represented individuals as balls when they repeated each other’s zig-escape. This representation, obviously, hides a change in orientation due to rolling. When individuals were represented by spheres, no agitation wave was observed in the form of a dark band while the escape manoeuvre of rolling sideward was transferred through the flock (compare Fig. 3f versus 3e, Movie S5 versus S4).

In empirical data of starling flocks, the wave speed varies between 3.66 and 25.24 m/s (Table 2) (Procaccini et al. 2011). Many factors may cause this variation. One may be the location of attack.

**Table 2:** Parameters generating similar speed of the agitation wave in the model as in empirical data from Procaccini et al. (2011). In the model: NND = average distance to nearest neighbours and Repetition range = number of neighbours screened for escape manoeuvres

Agitation waves			
Empirical	Model		
Speed (m/s)	Speed (m/s)	NND (m)	Repetition range
15.56	15.53	0.78	6
6.89	6.93	0.71	3
25.24	25.01	1.93	7
17.47	17.48	0.86	6
25.10	25.02	1.93	7
13.73	13.80	1.32	4
7.79	7.90	0.73	5
18.26	18.20	1.52	6
7.63	7.59	0.93	3
3.66	3.83	0.73	2
14.48	14.63	1.32	5
11.76	11.76	1.13	4
8.21	8.20	0.73	6
10.44	10.44	0.93	3
13.04	13.16	1.13	5

**Fig. 4:**
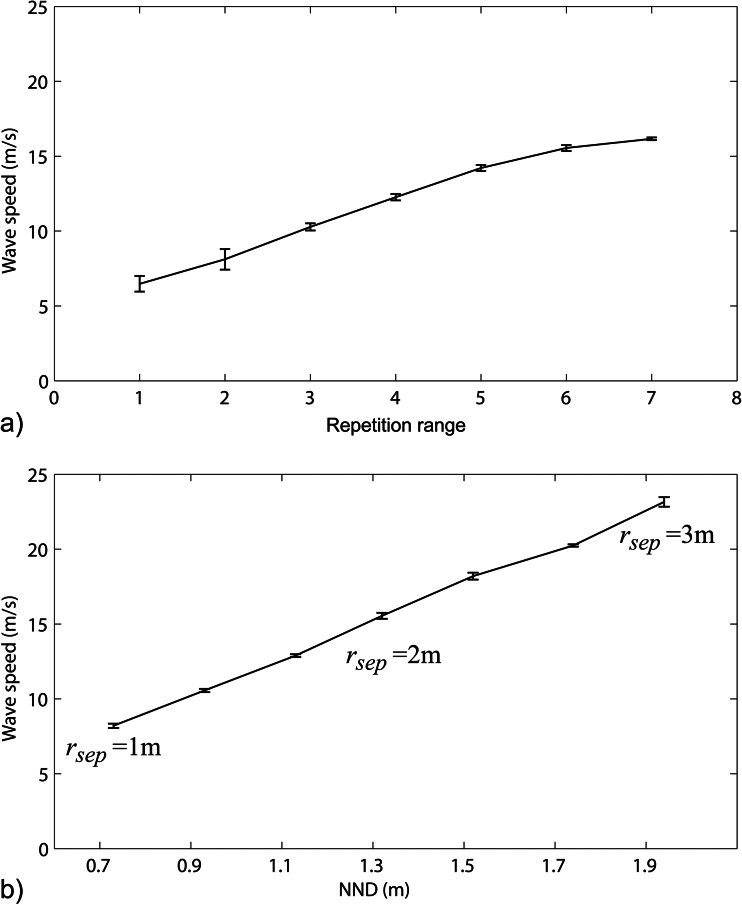
Speed of the wave (average and standard deviation) in the model and its dependence on **a** the range of repetition (the number of neighbours screened for an escape manoeuvre) and **b** the average distance to the nearest neighbour (NND), which was set using different values of the separation range, *r*_sep_

Regarding factors inducing wave speed, the range of repetition and flock density come to mind easily. When individuals screen a larger number of neighbours (indicating a larger range of repetition) for potential manoeuvres of danger, we expected the speed of the wave to increase. Indeed, extending the range of repetition from two till seven neighbours increased the speed of the wave in the model (Fig. 4a).

If a flock was less dense, meaning that on average individuals were further apart, wave speed was expected to be higher too because the reaction time remains the same (within the ranges of distances we tested, namely from 0.7 to 2 m). Empirically, flock density (measured as the average distance to the nearest neighbours) varies at least between 0.68 and 1.51 m (Ballerini et al. 2008b). Approximately within this range (Table 1), the model indeed showed that for a range of repetition of six neighbours transmission is faster if the flock is less dense (Fig. 4b).

These two parameters (range of repetition and sparseness of the flock) sufficed to generate the same range of speeds of waves as recorded in the empirical data for ranges of repeating escape manoeuvres from two till seven neighbours and for densities of the flocks between 0.71 and 1.93 m (Table 2). Unfortunately, empirical data for the wave events do neither include information about the range of repetition nor the average distance to the nearest neighbours (Table 2) (Procaccini et al. 2011).

## Discussion

We show that only if individuals repeat a ‘cue of danger’ an agitation wave results. It results not in the form of a density wave as has been assumed by us formerly (Procaccini et al. 2011). Instead, it results only if individuals repeat an escape manoeuvre related to changing their orientation, namely by rolling to one side (not both sides) during a single manoeuvre, the zig-escape. In this case, waves result that resemble agitation waves observed empirically in flocks of starlings (Procaccini et al. 2011).

In the model, the waves due to the zig-manoeuvre arise merely from the difference in orientation of the individuals towards the observer (and thus difference in the visibility of the wing area). Thus, when we see the flock from the side, the projection of the wing surface visible to us increases when birds have rolled sideward, and it decreases again when they fly level. As a consequence of the repetition of the escape manoeuvre by rolling, we see a dark band (of birds that are temporarily rolled sideward) moving away from the predator. There are no visible changes in density in the flock during an agitation wave (since no wave is visible if birds are represented as spheres).

The reasons why speeding up forward does not lead to a visible density wave may be twofold: individuals may not be coming sufficiently close to others (because of collision avoidance) and may be moving too slow to create visible changes in density (since rolling is faster) (Warrick 1998).

Agitation waves in flocks of dunlins and in schools of anchovies are due to changes of orientation also, but in contrast to our model in dunlins and anchovies, changes in orientation are associated with changes in colour (Radakov 1973; Potts 1984; Buchanan et al. 1988; Gerlotto et al. 2006). Besides in dunlins, the escape manoeuvre is really a full zigzag because the bird rolls to both sides in a single manoeuvre. Therefore, we alternatingly see the large projection of the dark dorsal side of the bird and the light ventral side. In case of a half zigzag, thus a mere zig, only the large projection of the ventral or dorsal side is exposed depending on whether the bird rolls left and back or right and back.

We show that no long-range interaction is needed (see Introduction). While keeping the distance to the nearest neighbours approximately within empirical ranges, we obtain in the model similar wave speeds as reported for empirical data when individuals are anticipating the escape manoeuvre of their two to seven closest neighbours. Thus, the range of interaction for repeating an escape reaction does not exceed the empirically established topological range of six or seven influential neighbours during coordination in the absence of an attack by a predator. This differs from what has been suggested for the waves of dunlins (Potts 1984) and of giant honeybees (Kastberger et al. 2008). However, the wave speed for dunlins (14.6 m/s) is similar to that of starlings (15 m/s). Therefore, even in dunlins, no long-range interaction is needed, in contrast to suggestions by Potts (Potts 1984). Our results confirm findings of waves of coordinated movement in emperor penguins (*Aptenodytes forsteri*). In a comparison between a mathematical model and empirical data, it was shown that a single step forward from a single penguin located in a densely packed huddle could trigger a complete wave (Gerum et al. 2013).

Wave speed in the model increases with the range of repetition representing the number of neighbours that each individual screens for mimicking their manoeuvres of escape (Fig. 4a) and also with the sparseness of the flock. There are probably more factors influencing speed of the wave. As expected, it decreases with increase of reaction time and cue identification and is not affected by flock size (Fig. S1). Other factors are outside the scope of this paper.

In our model, the repetition of escape manoeuvres was needed for waves to occur because otherwise the effect of the escape manoeuvre decayed too fast. A similar decay, called damping, was found for other models of moving groups (Cavagna et al. 2015), but during the turning of a real flock, remarkably, damping appeared to be absent (Attanasi et al. 2014).

As to the sensitivity of the wave phenomenon, the observation of an agitation wave due to a change in orientation of flock members is robust. We have shown that it remains for different values of the range of repeating, density, reaction time (data available on request), cue identification time (data available on request) and flock size.

Regarding the generality of our model, our model could be useful in analysing aspects of waves of dunlins (Potts 1984). For this, it should be adapted to the specifics of flying and flocking of dunlins. Our model is not helpful for different kinds of wave phenomena such as the waves that spread through flocks of semipalpated sandpipers, *Calidris pusilla*, when they depart (Beauchamp 2012). Here, related kinds of models are needed, such as models based on social facilitation (mimetic behaviour) where individuals are more likely to depart depending on the percentage and absolute number of individuals that have already departed (Pillot et al. 2011).

Although our model does neither represent realistically all the details of flying behaviour nor of coordination among birds, its flocking patterns resemble a large number of empirical patterns of flocking (Hemelrijk and Hildenbrandt 2012), such as flock shape, orientation and internal structure (related to density at front and back half, deviations of velocity (Hemelrijk and Hildenbrandt 2015) and diffusion in the flock (CKH and HH, unpublished data)). It is therefore useful for indicating where empirical data are lacking and in generating hypotheses. Lack of empirical data related to the occurrence of waves concerns the associated flock density, flock size, location of attack (back, front, top or bottom of the flock), the kind of attack (dive or flight pursuit), cue identification time and behavioural aspects of changing orientation during escape manoeuvres. Regarding the generation of hypotheses by the model, we here deliver seven new hypotheses. The first hypothesis is that agitation waves in starling flocks arise from rolling movements (specifically a zig, thus half a zigzag), secondly that a range of screening two till seven neighbours for their escape manoeuvres suffices, thirdly that wave speed increases with this range, fourthly with the sparseness of the flock, fifthly it decreases with increasing reaction time, sixthly with cue identification time and seventhly, it does not depend on flock size. These are hypotheses for empirical scientists to refute or confirm.

## Electronic supplementary material

Below is the link to the electronic supplementary material.

ESM 1 (DOCX 2501 kb)

Movie S1 The escape manoeuvre of rolling sideward, not repeated by others. This movie shows that this escape manoeuvre without being repeated by other (others merely adjust their movement direction) does not result in an agitation wave. (MP4 503 kb)

Movie S2 Repetition of the escape manoeuvre of speeding-up-forward repeated by neighbours. This movie shows that from repetition of this escape manoeuvre an agitation wave is not observed. (MP4 745 kb)

Movie S3 Repetition of the zigzag-escape manoeuvre of rolling to one side, back, rolling to the other side and back again (trajectory in Fig. 1). This movie shows that a double-banded agitation wave in StarDisplay arises from the repetition of the escape manoeuvre of sideward rolling and back. (MP4 900 kb)

Movie S4a Repetition of the zig-escape manoeuvre of sideward rolling and back (Fig. 1). This movie shows that a single-banded agitation wave in StarDisplay arises from the repetition of the escape manoeuvre of sideward rolling and back. (MP4 731 kb)

Movie S4b A dense flock and repetition of the escape manoeuvre of sideward rolling and back. This movie shows that in a dense flock in StarDisplay an agitation wave results from repetition of the escape manoeuvre of sideward rolling and back. Note that a preliminary predator is added. (MP4 154 kb)

Movie S5 A spherical body shape and repetition of the zig-escape manoeuvre of sideward rolling and back. This movie shows that the repetition of the zig-escape manoeuvre does not generate an observable agitation wave when the body shape is circular. (MP4 154 kb)
