# Supplementary material for: What underlies waves of agitation in starling flocks
Source: Behav Ecol Sociobiol. 2015 Mar 25;69(5):755–64. doi: 10.1007/s00265-015-1891-3 (PMC4564680; doi:10.1007/s00265-015-1891-3)
Supplement: Supplementary file 1 — (DOCX 2501 kb) [file 265_2015_1891_MOESM1_ESM.docx]

Supplementary material

# What underlies waves of agitation in starling flocks

Charlotte K. Hemelrijk^1*^, Lars van Zuidam^1^, Hanno Hildenbrandt^1^

^1^Behavioural Ecology and Self-organisation, Groningen Institute for Evolutionary Life Sciences, University of Groningen, Nijenborgh 7, 9747AG Groningen, The Netherlands

^*^Corresponding author: [c.k.hemelrijk@rug.nl](mailto:c.k.hemelrijk@rug.nl)

Behavioural Ecology and Self-organisation, GELIFES,

University of Groningen,

Nijenborgh 7,

9747AG Groningen,

The Netherlands

Tel 0031-503638084
Fax 0031-50-3633400

Here we describe

1. the results of wave speed related to reaction time, cue-identifiction time and flock size and

2. the model and its main parameters.

# 1. Wave speed and reaction time, cue-identification time and flock size





**Fig. S1**: Speed of the wave (average and standard deviation) in the model and its dependence on a) reaction time, b) cue-identification time and c) group size.

# 2. The basic model

## Representation of individuals

Each individual is characterized by its mass, *m*, its speed, *v*, and the location of its body, ***p***. Birds keep their head level in order to stabilize their perception and to isolate their visual and vestibular system from the wild movements of their body as their body rotates around the roll and axis (Figure 1) (Warrick et al. 2002). Therefore, we represent the orientation of the head $\boldsymbol{H}$ and the body $\boldsymbol{B}$ in separate local coordinate systems given by matrices $\boldsymbol{H}=\left[ \boldsymbol{h}_{\boldsymbol{x}}\boldsymbol{,}\boldsymbol{h}_{\boldsymbol{y}}\boldsymbol{,}\boldsymbol{h}_{\boldsymbol{z}} \right]$ and ***B*** =[***e_x_*, *e_y_*, *e_z_*]**.

Following the model by Reynolds (Reynolds 1987), the orientation of the body is indicated by its forward direction, ***e_x_***, its sideward direction, ***e_y_***, and its upward direction, ***e_z_***, which it changes by rotating around these three principal axes, ***e_x_***, ***e_y_*** and ***e_z_*** (*roll*, *pitch* and *yaw*) (Fig. S2).





**Fig. S2:** A bird with its three principal axes around which it can rotate: roll, pitch and yaw.

The orientation of the ‘head’-system, ***H*** is given by (Fig. S2):

$\boldsymbol{h}_{\boldsymbol{x}}=\boldsymbol{e}_{\boldsymbol{x}}$ (S1a)

$\boldsymbol{h}_{\boldsymbol{y}}=\frac{\boldsymbol{e}_{\boldsymbol{x}}\times{[0,0,1]}^{T}}{\boldsymbol{|e}_{\boldsymbol{x}}\times{[0,0,1]}^{T}|}$ (S1b)

$\boldsymbol{h}_{\boldsymbol{z}}=\boldsymbol{h}_{\boldsymbol{y}}\times\boldsymbol{e}_{\boldsymbol{x}}$ (S1c)

Where ‘×’ denotes the cross product.


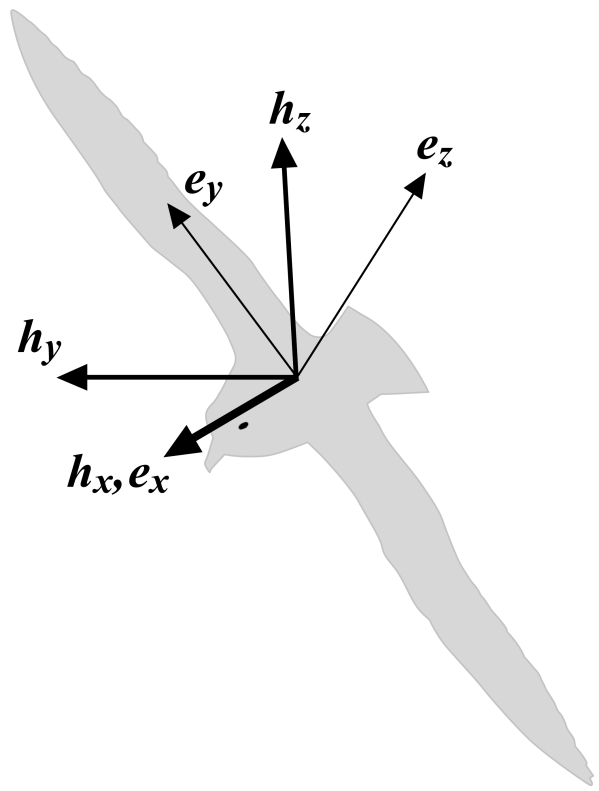


**Fig. S3:** Head-system **[*h_x_, h_y_, h_z_*]** and body-system **[*e_x_, e_y_, e_z_*]** of a bird.

## Field of view

The field of view of the individuals in the simulation is spherical with a wedge-shaped blind area at the back (Martin 2007). It is defined in the head-system (Fig. S4). Whether another individual *j* is in the field of view of an individual *i* depends on the azimuthal angle, $\varphi_{ij}$ of the position of individual *j* in the head-system of individual *i,* ***p’*** :

$\boldsymbol{p'=}\boldsymbol{(p}_{\boldsymbol{j}}\boldsymbol{-}\boldsymbol{p}_{\boldsymbol{i}}\boldsymbol{)}\boldsymbol{H}_{\boldsymbol{i}}$ The position of *j* in the head-system of *i* (S2a)

$\varphi_{ij}=arctan\left( {p_{y}^{'}}/{p_{x}^{'}} \right)$ The azimuthal angle of *j* in head system of *i* (S2b)

$\left| \varphi_{ij}-{180}^{o} \right|<\varphi_{b}/2$; Individual *j* not in the blind angle of *i* (S2c)





**Fig. S4:** Field of view in head-system. a) View from aside and above. b) Top view.

## Reaction time

The reaction time of an individual, or the latency period until the bird updates its environment, *U,* is initially randomly drawn from a normal distribution with mean *μ_u_* and standard deviation *σ_u_*. Subsequently, every time step and for each individual, it is adjusted by adding a small value *ζ_u_*(t) drawn from of a uniform random distribution in the range [*-ζ_u_ ,+ζ_u_*] (Table 1):

$U=normrnd(\mu_{u},\sigma_{u})$ Normal distribution of reaction time (S3a)

$u\left( t \right)=U+\zeta_{u}(t)$ Actual reaction time (S3b)

## Influential neighbours or topological interaction

To represent that individuals interact on average with a constant number of their closest neighbours (i.e. topological interaction), each individual *i* in the model adapts its metric search radius, *R_i_*(t) (Hemelrijk and Hildenbrandt 2008) as follows:

$R_{i}(t+u(t))=\left( \left( 1-s \right)+s\cdot\sqrt[3]{\frac{n_{c}}{\left| N_{i}(t) \right|}} \right){\cdot R}_{i}(t)$ Adaptive interaction range (S4a)

$N_{i}≝\left\{ j\epsilon N; d_{ij}\leq R_{i}; j\neq i; j not in blind are of i \right\}$ Neighborhood of *j* (S4b)

where *u*(*t*) is the reaction time (Equ. S4b), *s* is an interpolation factor, *N_i_*(*t*) is the neighbourhood of individual *i* at time *t*, i.e. the set of influential neighbours of an individual *i* which is composed of |*N_i_*(*t*)| neighbours from the total flock of size *N*, *n_c_* is the fixed number of topological interaction partners and *d_ij_* is the distance between individual *i* and *j* given by |***p_j_*** – ***p_i_***|, where ***p_i_*** denotes the position of an individual *i*. Thus, the radius of interaction at the next step in reaction-time, *R_i_*(*t*+*u*), increases if the number of interaction partners |*N_i_*(*t*)| is smaller than the targeted number *n_c_*, and decreases if it is larger; it remains as before if |*N_i_*(*t*)| equals *n_c_*. Here *R_i_* cannot decrease below the minimal radius *r_h_* (representing the wing span, also referred to as hard sphere (Ballerini et al. 2008)) in which individuals maximally avoid each other. The interpolation factor *s* determines the step-size of the changes and herewith, the variance of the number of actual influential neighbours.

## Steering force

## Social forces

The individuals are led by the three social behaviours: separation, cohesion and alignment. These are represented as social forces (Helbing and Molnar 1995). Separation and cohesion depend on the average direction of the influential neighbours $\boldsymbol{d}_{ij}$:

$\bar{\boldsymbol{d}_{ij}}=\frac{1}{\left| N_{i}(t) \right|}\sum_{j\in N_{i}(t)} \frac{\boldsymbol{d}_{\boldsymbol{ij}}}{\left| \boldsymbol{d}_{\boldsymbol{ij}} \right|}$ (S5)

where $\boldsymbol{d}_{\boldsymbol{ij}}\boldsymbol{=(}\boldsymbol{p}_{\boldsymbol{j}}\boldsymbol{-}\boldsymbol{p}_{\boldsymbol{j}}\boldsymbol{)}$ is the vector pointing from individual *i* to its neighbor *j*. To smooth the effect of distance on separation and cohesion at the range between $r_{h}$ and $r_{sep}$, the so called *smootherstep* sstep(x) is applied:

$x=\left\{ \begin{aligned} 0, d_{ij}\leq r_{h} \\ 1, d_{ij}\geq r_{sep} \\ (d_{ij}-r_{h})/(r_{sep}-r_{h}), otherwise \end{aligned} \right.$ (S6a)

$\mathrm{sstep}\left( x \right)=6x^{5}-15 x^{4}+10 x^{3}$ (S6b)

which is chosen because it interpolates the values smoothly. Here $r_{h}$is the radius of the hard sphere and $r_{sep}$ is the so-called separation radius (Hildenbrandt et al. 2010). The separation force is given by:

$\boldsymbol{f}_{s}'=-\frac{w_{s}}{\left| N_{i}(t) \right|}\sum_{j\in N_{i}(t)} \left( 1-\mathrm{sstep}\left( d_{ij} \right) \right)\frac{\boldsymbol{d}_{\boldsymbol{ij}}}{\left| \boldsymbol{d}_{\boldsymbol{ij}} \right|}$ Separation (S7a)

$\boldsymbol{f}_{s}=\boldsymbol{H}\boldsymbol{f}_{\boldsymbol{s}}\boldsymbol{'H}$ Separation (head-system) (S7b)

and the cohesion force is given by:

$\boldsymbol{f}_{c}'=\frac{w_{c}\cdot\left| \bar{\boldsymbol{d}_{\boldsymbol{ij}}} \right|^{2}}{\left| N_{i}(t) \right|}\sum_{j\in N_{i}(t)} \mathrm{sstep}\left( d_{ij} \right)\frac{\boldsymbol{d}_{\boldsymbol{ij}}}{\left| \boldsymbol{d}_{\boldsymbol{ij}} \right|}$ Cohesion (S8a)

$\boldsymbol{f}_{\boldsymbol{c}}\boldsymbol{=H}\boldsymbol{f}_{\boldsymbol{c}}^{\boldsymbol{'}}\boldsymbol{H}$ Cohesion (head-system) (S8a)

where $w_{s}$ and $w_{c}$ are weighting factors (Table 1). $\bar{\boldsymbol{d}_{ij}}$ of Equ. S5 gives the average direction of the neighbour set, the vector of the local circularity (Hemelrijk and Hildenbrandt 2011)*.* The magnitude of $\bar{\boldsymbol{d}_{ij}}$ inside a flock is close to zero and at its periphery is close to one (Hemelrijk and Wantia 2005). Note that $\bar{\boldsymbol{d}_{ij}}$ differs here from our former equation for circularity in that it is more animal-centred because it does not consider neighbours in the blind area. It represents the extra tendency of individuals at the periphery of the flock to move inwards. This represents the strong tendency of real birds at the flock border to avoid the risk of predator attacks from the outside (Hamilton 1971). This addition to the model causes the border of the flock to become sharp like in real birds (Ballerini et al. 2008).

As for alignment, we assume in the model that a bird aligns both its heading to that of its neighbours and its spatial orientation. In order to align its heading to the average heading of its neighbours, an individual experiences the force,$\boldsymbol{f}_{ah}$:

$\boldsymbol{f}_{ah}=\frac{w_{ah}}{\left| N_{i}(t) \right|}\sum_{j\in N_{i}(t)} \boldsymbol{e}_{\boldsymbol{xj}}-\boldsymbol{e}_{\boldsymbol{xi}}$ Alignment of heading (S9)

Here, $\boldsymbol{e}_{\boldsymbol{xi}}$ and $\boldsymbol{e}_{\boldsymbol{xj}}$ are vectors indicating the forward direction of individuals *i* and *j* and *w_ah_* is the weighting factor for alignment of heading (Table 1). In order to align the banking angle to that of its neighbours, an individual experiences a force, $\boldsymbol{f}_{ab}$ , represented by a vector along the wing axis that induces roll:

$\boldsymbol{f}_{ab}=-\boldsymbol{e}_{\boldsymbol{yi}}\frac{w_{ab}}{\left| N_{i}(t) \right|}\sum_{j\in N_{i}(t)} \boldsymbol{e}_{\boldsymbol{yj}}\boldsymbol{\cdot}\boldsymbol{e}_{\boldsymbol{yi}}$ Alignment of banking (S10)

Here, $\boldsymbol{e}_{\boldsymbol{yi}}$ and $\boldsymbol{e}_{\boldsymbol{yj}}$ are the vectors indicating the side direction (wing axis) of individuals *i* and *j* and *w_ab_* is the weighting factor for alignment of banking (Table 1).

The total social force is given by the sum of Equ. S7-10:

$\boldsymbol{F}_{\boldsymbol{social}}=\boldsymbol{f}_{\boldsymbol{s}}+\boldsymbol{f}_{\boldsymbol{c}}+\boldsymbol{f}_{\boldsymbol{ah}}+\boldsymbol{f}_{\boldsymbol{ab}}$ Social force (S11)

## Speed control

As to its speed, a force, $\boldsymbol{f}_{\boldsymbol{\tau}}$, (Equ. S12) brings an individual back to its cruise speed *v_0_* after it has deviated from it (Hemelrijk and Hildenbrandt 2008):

$\boldsymbol{f}_{\boldsymbol{\tau}}=\frac{m}{\tau}\left( v_{0}-v \right) \boldsymbol{e}_{\boldsymbol{x}}$ Speed control (S12)

where *τ* represents the relaxation time, *m* is the mass of the individual *i* and $v_{0}$ its cruise speed, $v$ its current speed and $\boldsymbol{e}_{\boldsymbol{x}}$ its forward direction.

## Attraction to roost

Individuals of a flock fly at a similar height above the roost (the site where the birds sleep), because we made them experience both in a horizontal and vertical direction a force of attraction to the ‘roosting area’, $\boldsymbol{F}_{\boldsymbol{Roost}}$, (Equ. S13). The strength of the horizontal attraction, $\boldsymbol{f}_{\boldsymbol{RoostH}}$, is greater, the more radially it moves away from the roost; it is weaker if it is already returning. The sign in Equ. S13b is chosen such that it reduces the outward heading and ***n*** is an outward pointing vector normal to the boundary of the roost. The actual direction of the horizontal attraction force is given by $\boldsymbol{e}_{\boldsymbol{y}}$ which is the individual’s lateral direction. Vertical attraction, $\boldsymbol{f}_{\boldsymbol{RoostV}}$, is proportional to the vertical distance from the preferred height, $d_{alt}$, above the roost, $w_{RoostH}$ and $w_{RoostV}$ are weighting factors.

$\boldsymbol{F}_{\boldsymbol{Roost}}=\boldsymbol{f}_{\boldsymbol{RoostH}}+\boldsymbol{f}_{\boldsymbol{RoostV}}$ Attraction to roost (S13a)

$\boldsymbol{f}_{\boldsymbol{RoostH}}=\pm w_{RoostH}\left( \frac{1}{2}+\frac{1}{2}\left( \boldsymbol{e}_{\boldsymbol{x}}\boldsymbol{\cdot}\boldsymbol{n} \right) \right)\cdot\boldsymbol{e}_{\boldsymbol{y}}$ Horizontal attraction to roost (S13b)

$\boldsymbol{f}_{\boldsymbol{RoostV}}=-w_{RoostV}\left( d_{alt} {\cdot\left[ 0,0,1 \right]}^{T} \right)$ Vertical attraction to roost (S13b)

## Random noise

Errors in perception and behaviour (caused by time used in cognitive processing, deciding and preparing and actualising motor output) are incorporated in two ways, through the delayed and asynchronous reaction of individuals to their environment (due to their reaction time) and by adding a random force. The reaction time (76ms) represents the delay with which individuals respond to their environment and is updated asynchronously and less frequently than the physics in the model (1ms) (Table S1). The random force indicates unspecified stochastic influences (Equ. S14) with *ξ* being a random unit vector from a uniform distribution and *w_ξ_* being a fixed scaling factor.

$\mathbf{f}_{\xi_{_{\mathbf{i}}}}=w_{\xi}\cdot\mathbf{\xi}$ Random force (S14)

The sum of the social force, the speed control and the random force is labelled as ‘steering force’ (Equ. S15).

$\boldsymbol{F}_{\boldsymbol{steering}}={\boldsymbol{F}_{\boldsymbol{social}}+\boldsymbol{f}_{\boldsymbol{\tau}}+\boldsymbol{F}}_{\boldsymbol{Roost}}+\boldsymbol{f}_{\boldsymbol{\xi}}$ Steering force (S15)

The magnitude of the steering force is restricted to its maximum $F_{max}$ (Table S1).

# Flight model

The flight model is based on fixed wing aerodynamics, i.e. *the lifting line theory for elliptical wings* (Taylor and Thomas 2014). The three basic equations are:

$F=\frac{1}{2}\rho v^{2}SC_{F}$ Magnitude of aerodynamic force (S16a)

$L=\frac{1}{2}\rho v^{2}SC_{L}$ Magnitude of lift (S16b)

$D=\frac{1}{2}\rho v^{2}SC_{D}$ Magnitude of induced drag (S16c)

where $\rho$ is the air density, *v* is the air speed and *S* the wing area of the bird. The lift coefficient, *C_L_*, and the lift-drag ratio, *C_L_/C_D_* , are approximated for steady glide as:

$C_{L}=\frac{2\pi\alpha}{1+\frac{2}{AR}+16(\log\left( \pi AR \right)-\frac{9}{8})/{(\pi AR)}^{2}}$ Lift coefficient (S17a)

$\frac{C_{L}}{C_{D}}=\frac{\pi}{C_{L}} AR$ Lift-drag ratio (S17b)

where *AR* is the aspect ratio of the wing and $\alpha$ is the angle of attack of the wing.

The equations for the flight model are:

$\boldsymbol{L}=L \boldsymbol{e}_{\boldsymbol{z}}$ Lift force (S18a)

$\boldsymbol{D}=-D \boldsymbol{e}_{\boldsymbol{x}}$ Drag force (S18b)

$\boldsymbol{T}_{\boldsymbol{0}}=D(v_{0}) \boldsymbol{e}_{\boldsymbol{x}}$ Default thrust at cruise speed *v_0_* (S18c)

$\boldsymbol{W}=mg \left[ 0,0,-1 \right]^{T}$ Weight (S18c)

Where $D(v_{0})$ represents the drag at cruise speed, $v_{0}$, *g* is gravitation constant, *m* is mass of the individual.

The flight force is given by:

$\boldsymbol{F}_{\boldsymbol{Flight}}=\boldsymbol{L}+\boldsymbol{W}+\boldsymbol{T}_{\boldsymbol{0}}+\boldsymbol{D}$ Flight force (S19)

The flight force is calculated every *dt* seconds to represent the continuity of physical forces. This update frequency is much higher than that of the steering force (about 100 times as often), since updates of steering forces depend on reaction time of the bird (Table S1).

# Integration

To calculate new position and velocity, Verlet integration is used instead of Euler integration (Hildenbrandt et al. 2010, Hemelrijk and Hildenbrandt 2011), because of its greater precision:

$\boldsymbol{F}\left( t+dt \right)=\boldsymbol{F}_{\boldsymbol{steering}}+\boldsymbol{F}_{\boldsymbol{Flight}}$ Total force (S20a)

$\boldsymbol{v}\left( t+\frac{dt}{2} \right)=\boldsymbol{v}\left( t \right)+\boldsymbol{a}\left( t \right)dt/2$ Half step velocity (S20b)

$\boldsymbol{p}\left( t+dt \right)=\boldsymbol{p}\left( t \right)+\boldsymbol{v}(t+\frac{dt}{2})$ Position (S20c)

$\boldsymbol{a}\left( t+dt \right)=\boldsymbol{F}(t+dt)/m$ Acceleration (S20d)

$\boldsymbol{v}\left( t+dt \right)=\boldsymbol{v}\left( t+\frac{dt}{2} \right)+\frac{\boldsymbol{a}\left( t+dt \right)dt}{2}$ Velocity (S20e)

# Roll and pitch

In order to perform a turn an individual redirects its lift by rolling its body around the forward axis until the lateral component of the lift equals the lateral component of the steering force (Fig. S5). This results in a so called banked turn that resembles empirical data in that individuals lose height during turns and that they roll into the turn faster than that they roll back (Gillies et al. 2011). The roll angle is relative towards the horizontal, and the horizontal is given by ***h_y_***. The difference between the lateral component of the steering force $F_{sl}$and of the lift $L_{l}$ leads to the angular speed as follows:

$F_{sl}=\boldsymbol{F}_{\boldsymbol{Steering}}\cdot\boldsymbol{h}_{\boldsymbol{y}}$ Lateral component of steering force (S21a)

$L_{l}=\boldsymbol{L}\cdot\boldsymbol{h}_{\boldsymbol{y}}$ Lateral component of lift force (S21b)

$\omega_{r}={d\beta}/{dt}=w_{r}\left( F_{sl}-L_{l} \right)$ Angular speed around roll axis, $d\beta\ll1$ (S21c)

where $\beta$ is the banking angle and $w_{r}$ is a scaling factor (TableS1). Pitch is modeled by rotating around the pitch axis, $\boldsymbol{e}_{\boldsymbol{y}}$. In the model pitch is a consequence of a vertical component of the steering force of the body system, $F_{sv}$:

$F_{sv}=\boldsymbol{F}_{\boldsymbol{Steering}}\cdot\boldsymbol{e}_{\boldsymbol{2}}$ Vertical component of steering force (S22a)

$\omega_{p}={d\gamma}/{dt}=w_{p} F_{sv}$ Angular speed around pitch axis, $d\gamma\ll1$ (S22b)

Where $\gamma$represents the angle of pitching and $w_{p}$ is a scaling factor (Table S1).





**Fig. S5:** Rotation of the body system around the roll axis (facing towards the reader) in the situation where the lateral component of the lift, $L_{l}\cdot\boldsymbol{h}_{\boldsymbol{y}}$, equals the lateral component of the steering force, $F_{sl}\cdot\boldsymbol{h}_{\boldsymbol{y}}$ (Equ. S21).

# Rotation of the body system

Every integration time step roll and pitch are applied to the body system and renormalized with respect to the forward direction:

$\boldsymbol{e}_{\boldsymbol{x}}=\left( \boldsymbol{e}_{\boldsymbol{x}}+\omega_{p}\boldsymbol{e}_{\boldsymbol{z}} dt \right)/\left| \boldsymbol{e}_{\boldsymbol{x}}+\omega_{p}\boldsymbol{e}_{\boldsymbol{z}} dt \right|$ Corrected forward axis (application of pitch) (S23a)

$\boldsymbol{e}_{\boldsymbol{z}}'=\left( \boldsymbol{e}_{\boldsymbol{z}}+\omega_{r}\boldsymbol{e}_{\boldsymbol{y}} dt \right)/\left| \boldsymbol{e}_{\boldsymbol{z}}+\omega_{r}\boldsymbol{e}_{\boldsymbol{y}} dt \right|$ Application of roll (S23a)

$\boldsymbol{e}_{\boldsymbol{y}}=\left( \boldsymbol{e}_{\boldsymbol{x}}\times\boldsymbol{e}_{\boldsymbol{z}}\boldsymbol{'} \right)/\left| \boldsymbol{e}_{\boldsymbol{x}}\times\boldsymbol{e}_{\boldsymbol{z}}\boldsymbol{'} \right|$ Corrected side axis (S23c)

$\boldsymbol{e}_{\boldsymbol{z}}\boldsymbol{=}\boldsymbol{e}_{\boldsymbol{y}}\boldsymbol{\times}\boldsymbol{e}_{\boldsymbol{x}}$ Corrected up axis (S23d)

$\boldsymbol{v}=v \boldsymbol{e}_{\boldsymbol{x}}$ Corrected velocity (S23e)

where ‘×’ denotes the cross product.

| **Parameter** | **Description** | **Default value** |
| --- | --- | --- |
| *dt* | Integration time step | 1 ms |
| *Δu* | Average reaction time | 76 ms (Pomeroy and Heppner 1977) |
| *σ_u_* | std. deviation of reaction time | 10 ms (Videler 2005) |
| *v_0_* | Cruise speed | 10 m/s (Videler 2005) |
| *m* | Mass | 0.08 kg (Videler 2005) |
| *S* | Wing area | 48 cm^2^ (Videler 2005) |
| *AR* | Wing aspect ratio | 8.33(Videler 2005) |
| *α* | Angle of attack | 1^o^ |
| $\tau$ | Speed control | 10 s |
| *w_r_* | Roll control | 4 rad/s |
| *w_p_* | Pitch control | 1 rad/s |
| *n_c_* | Topological range | 6.5 |
| *s* | Interpolation factor | 0.1 *Δu* |
| *r_h_* | Radius of max. separation (“hard sphere”) | 0.2 m (Ballerini et al. 2008) |
| *r_sep_* | Separation radius (default) | 2 m |
| *w_s_* | Weighting factor separation force | 1 N |
| *ϕ* | Rear “blind angle” cohesion & alignment | 36°(Martin 1986) |
| *w_ah_* | Weighting factor alignment force (heading) | 2 N |
| *w_ab_* | Weighting factor alignment force (banking) | 2 N |
| *w_c_* | Weighting factor cohesion force | 1 N |
| *w_ξ_* | Weighting factor random force | 0.01 N |
| *w_RoostH_* | Weighting factor horizontal boundary force | 0.01 N/m |
| *w_RoostV_* | Weighting factor vertical boundary force | 0.005 N/m |

Table S1 Model parameters.

## References

Ballerini M, Cabibbo N, Candelier R et al (2008) Empirical investigation of starling flocks: a benchmark study in collective animal behaviour. Anim  Behav 76:201-215

Gillies JA, Thomas ALR, Taylor GK (2011) Soaring and manoeuvring flight of a steppe eagle Aquila nipalensis. J Avian Biol 42:377-386

Hamilton WD (1971) Geometry for the selfish herd. Journal of theoretical Biology 31:295-311

Helbing D, Molnar P (1995) Social Force Model for Pedestrian Dynamics. Physical Review E 51:4282-4286

Hemelrijk CK, Hildenbrandt H (2011) Some causes of the variable shape of flocks of birds. PLoS ONE 6:e22479

Hemelrijk CK, Wantia J (2005) Individual variation by self-organisation: a model. Neuroscience & Biobehavioral Reviews 29:125-136

Hemelrijk CK, Hildenbrandt H (2008) Self-organized shape and frontal density of fish schools. Ethology 114:245-254

Hildenbrandt H, Carere C, Hemelrijk CK (2010) Self-organized aerial displays of thousands of starlings: a model. Behav Ecol 21:1349-1359 doi:10.1093/beheco/arq149

Martin GR (1986) The Eye of a Passeriform Bird, the European Starling (Sturnus-Vulgaris) - Eye-Movement Amplitude, Visual-Fields and Schematic Optics. J of Comp Phys a-Sensory Neural and Behavioral Physiology 159:545-557

Martin GR (2007) Visual fields and their functions in birds. J of Ornith 148:S547-S562

Pomeroy H, Heppner F (1977) Laboratory determination of startle reaction time of the starling (Sturnus vulgaris). Anim Behav 25:720-725

Reynolds CW (1987) Flocks, Herds and Schools: A Distributed Behavioral Model. In: Proceedings of the 14th Annual Conference on Computer Graphics and Interactive Techniques. ACM, New York, pp 25-34

Taylor G, Thomas A (2014) Evolutionary Biomechanics. Selection, Phylogeny and Contraint. Oxford University Press, Oxford

Videler JJ (2005) Avian flight. Oxford University Press, Oxford

Warrick D, Bundle M, Dial K (2002) Bird maneuvering flight: Blurred bodies, clear heads. Integr Comp Biol 42:141-148
